# Supplementary material for: Viral N protein hijacks deaminase-containing RNA granules to enhance SARS-CoV-2 mutagenesis
Source: EMBO J. 2024 Nov 20;43(24):6444–68. doi: 10.1038/s44318-024-00314-y (PMC11649915; doi:10.1038/s44318-024-00314-y)
Supplement: Supplementary file 1 — Appendix [file 44318_2024_314_MOESM1_ESM.pdf]

Appendix

Viral N protein hijacks deaminase-containing RNA granules to enhance SARS-CoV-2 mutagenesis

Zhean Li, Lingling Luo, Xiaohui Ju, Shisheng Huang, Jia Liu, Pumin Zhang, Tian Chi, Peixiang Ma, Cheng Huang, Xingxu Huang, Qiang Ding, Yu Zhang

Table of contents

Pages 1-2: Appendix Table S1 to S2

Appendix Table S1. sgRNAs used in this study.

sgRNA Name	sgRNA sequence
G3BP1	CAGCGGGATCAAAGAGTGCG
G3BP1	AACGTTTGTCCTTGCTCCTG
G3BP2	AGAGTCGAAGCTAAACCAGA
G3BP2	CGCATCAATACCAAGGGTGT

Appendix Table S2. Primer sequences used in real time PCR.

Gene	Forward primer	Reverse primer
N protein-1	GGGGAACTTCTCCTGCTAGAAT	CAGACATTTTGCTCTCAAGCTG
N protein-2	TAATCAGACAAGGAACTGATTA	CGAAGGTGTGACTTCCATG
N protein-3	GACCCCAAAATCAGCGAAAT	TCTGGTTACTGCCAGTTGAATCTG

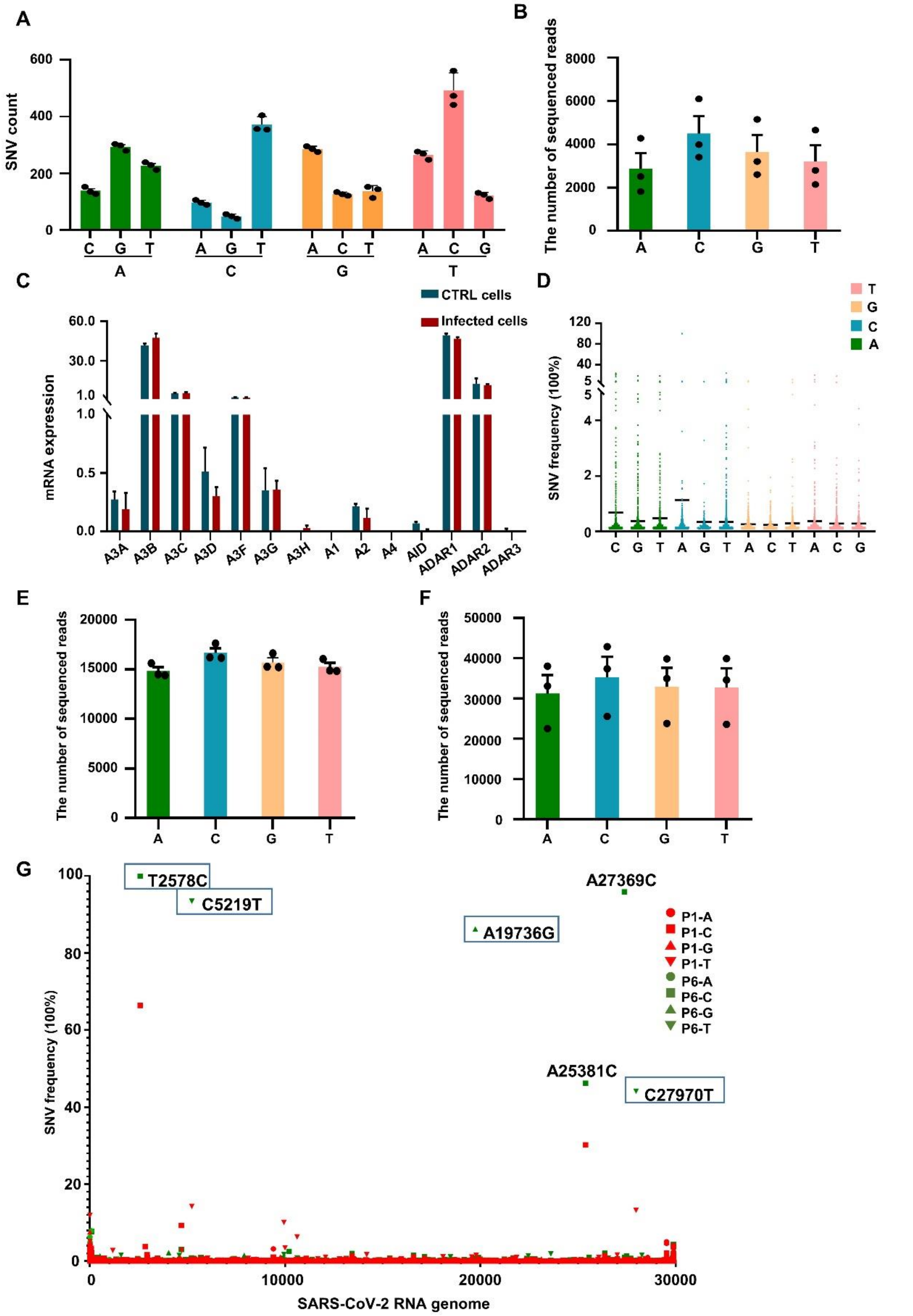

Appendix Figure S1. Host deaminases-mediated RNA editing is involved in host responses to SARS-CoV-2 infections. (A) The number of SNVs identified in SARS-CoV-2 transcriptomes utilizing a SARS-CoV-2 N protein-based genetic complementation system (allelic fraction ≥0.1%). Values and error bars were represented as the mean ± SEM of three independent biological replicates. **(B)** Average sequencing read count in SARS-CoV-2 transcriptomes. Values and error bars were

represented as the mean ± SEM of three independent biological replicates. **(C)** mRNA expression level of deaminases in Caco-2 cells with or without SARS-CoV-2 infection as determined by RNA sequencing. Values and error bars were represented as the mean ± SEM of three independent biological replicates. **(D)** Allelic fraction of the indicated SNVs identified in SARS-CoV-2 transcriptomes using a SARS-CoV-2 N protein-based genetic complementation system (allelic fraction ≥ 0.02%). Values and error bars were represented as the mean ± SEM of allelic fraction from the indicated SNVs in three independent biological replicates. **(E-F)** Average sequencing read count in SARS-CoV-2 transcriptomes at passage one (E) and six (F). Values and error bars were represented as the mean ± SEM of three independent biological replicates. **(G)** Distribution of SNVs across the SARS-CoV-2 genome using a SARS-CoV-2 N protein-based genetic complementation system after one and six passages (P1 and P6, allelic fraction ≥ 0.1%). Values and error bars were represented as the mean ± SEM of three independent biological replicates.

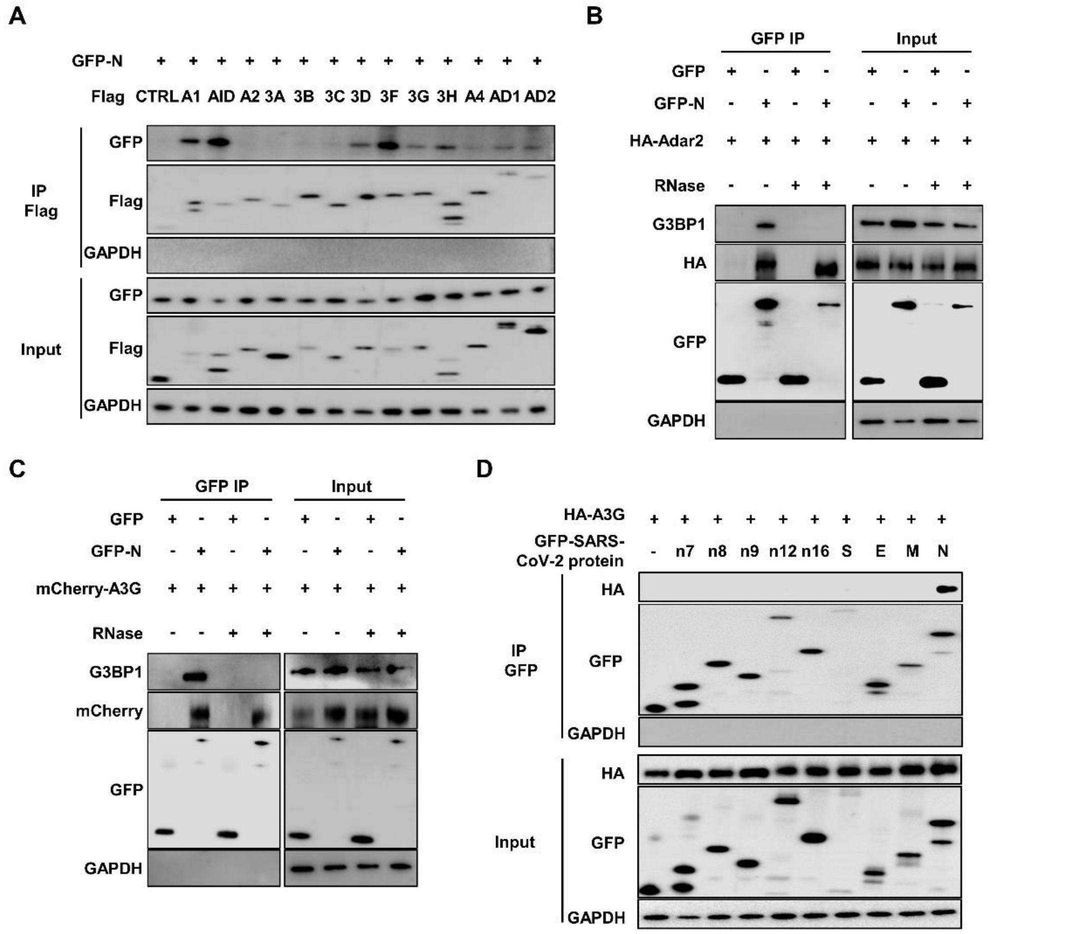

Appendix Figure S2. The SARS-CoV-2 N protein specifically interacts with host deaminases. **(A)** The SARS-CoV-2 N protein interacts with host deaminases. HeLa cells were co-transfected with plasmids encoding GFP-tagged N protein and Flag control or Flag-tagged host deaminases, including A1, AID, A2, A3A (3A), A3B (3B), A3C (3C), A3D (3D), A3F (3F), A3G (3G), A3H (3H) (3A-3D and 3F-3H), A4, ADAR1 (AD1) and ADAR2 (AD2). Cell lysates were subjected to immunoprecipitation using an anti-Flag antibody, and the associated proteins were then analyzed

by western blotting. **(B-C)** The N protein interacts with ADAR2 (B) or A3G (C) in an RNA-independent manner. HeLa cells transfected with GFP-tagged N protein and HA-tagged ADAR2 or mCherry-tagged A3G were subjected to immunoprecipitation with an anti-GFP antibody in the presence or absence of 100 μg/mL RNase A. The co-precipitation of ADAR2 or A3G was detected by western blotting using anti-HA and mCherry antibodies. **(D)** A3G specifically interacts with the SARS-CoV-2 N protein. HeLa cells were co-transfected with plasmids encoding HA-tagged APOBEC3G and GFP control or GFP-tagged SARS-CoV-2 genes, including nsp7 (n7), nsp8 (n8), nsp9 (n9), nsp12 (n12), nsp16 (n16), spike protein (S), envelope protein (E), membrane protein (M) and nucleocapsid protein (N). Cell lysates were subjected to immunoprecipitation using an anti-GFP antibody, and the associated proteins were analyzed by western blotting.

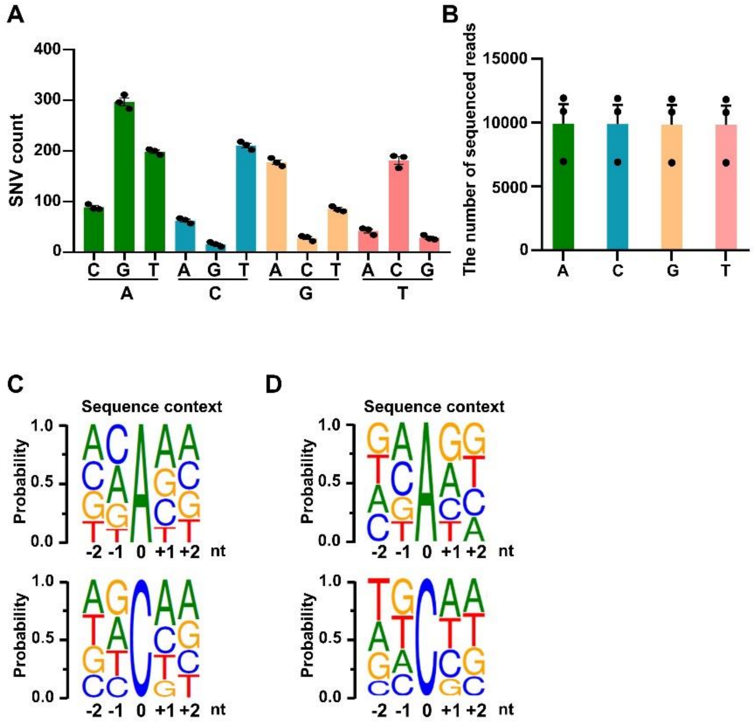

Appendix Figure S3. Host deaminases mediate RNA mutation in SARS-CoV-2 N protein mRNA. (A) The number of nucleotide changes in the N protein mRNA in HeLa cells. Cells were transfected with the N protein mRNA, lysed using Trizol reagent, and subjected to mRNA isolation and sequencing. Values and error bars were represented as the mean ± SEM of three independent biological replicates (allelic fraction ≥ 0.02%). **(B)** Average sequencing read count in the N protein mRNA. Values and error bars were represented as the mean ± SEM of three independent biological replicates. **(C)** Sequence contexts for A>I (G) and C>U (T) edited sites in the N protein transcriptome. **(D)** Sequence contexts for A>I (G) and C>U (T) edited sites in the N gene of the SARS-CoV-2 viral transcriptome.

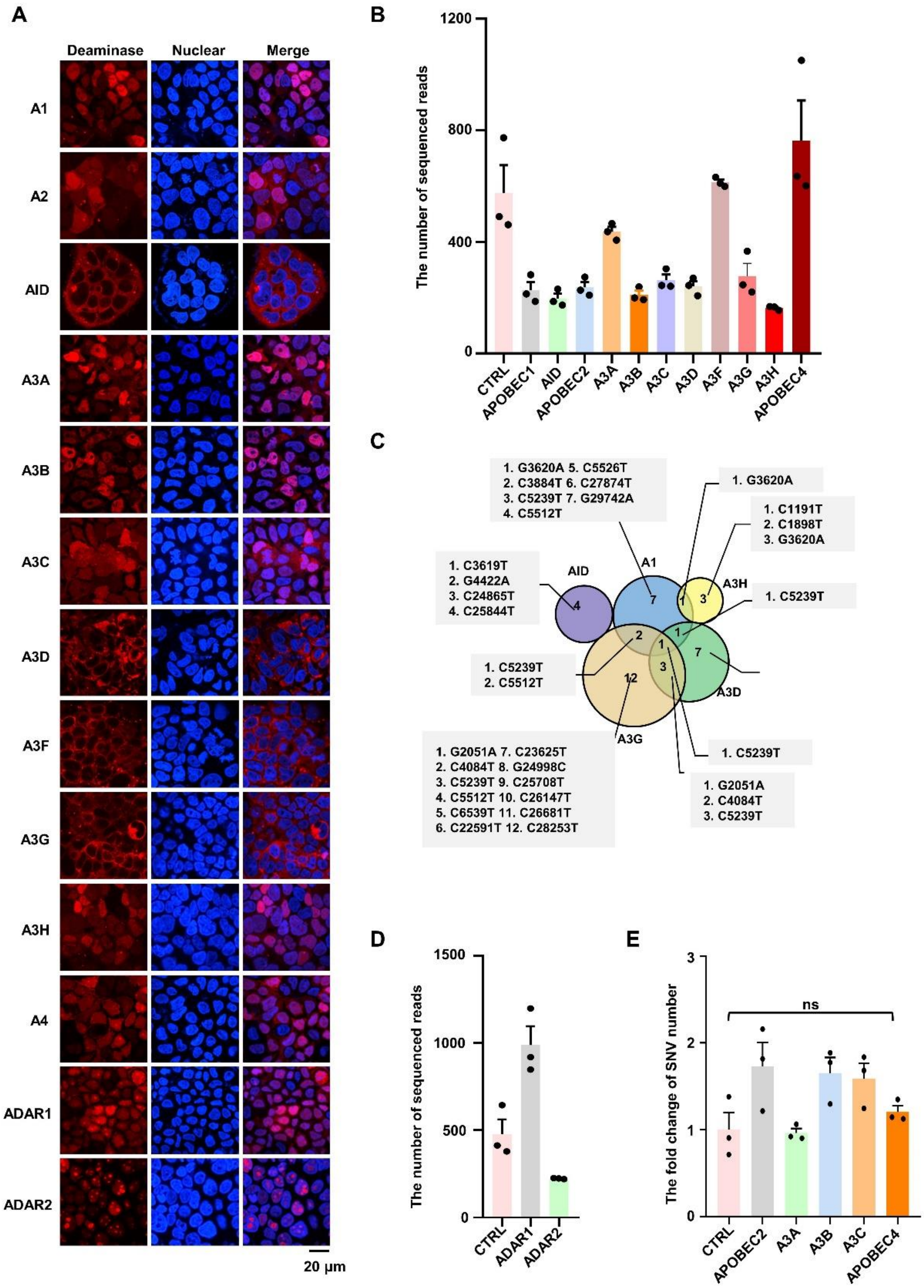

Appendix Figure S4. Host deaminases-mediated RNA editing in SARS-CoV-2 RNA mutation and production. (A) Expression of deaminases in Caco-2 cells. Stable cell lines expressing various deaminases were established through lentiviral transduction. **(B)** Average sequencing read count for C/G mutations in SARS-CoV-2 transcriptomes from deaminase-overexpressing Caco-2 cells. Values and error bars were represented as the mean ± SEM of three independent biological replicates. **(C)** Venn diagram illustrating various deaminases-induced viral mutation appeared in Delta and Omicron subclinical groups. **(D)** Average sequencing read count for A/T mutations in SARS-CoV-

2 transcriptomes from deaminase-overexpressing Caco-2 cells. Values and error bars were represented as the mean ± SEM of three independent biological replicates. **(E)** APOBECs deficient in their ability to interact with the N protein failed to increase SARS-CoV-2 RNA mutation (allelic fraction ≥2%). Values and error bars were represented as the mean ± SEM of three independent biological replicates. The mutation number of C>T/G>A was normalized to the number of control group. Statistical analysis was performed with a two-tailed unpaired t-test. ns > 0.05. ns, no significant.

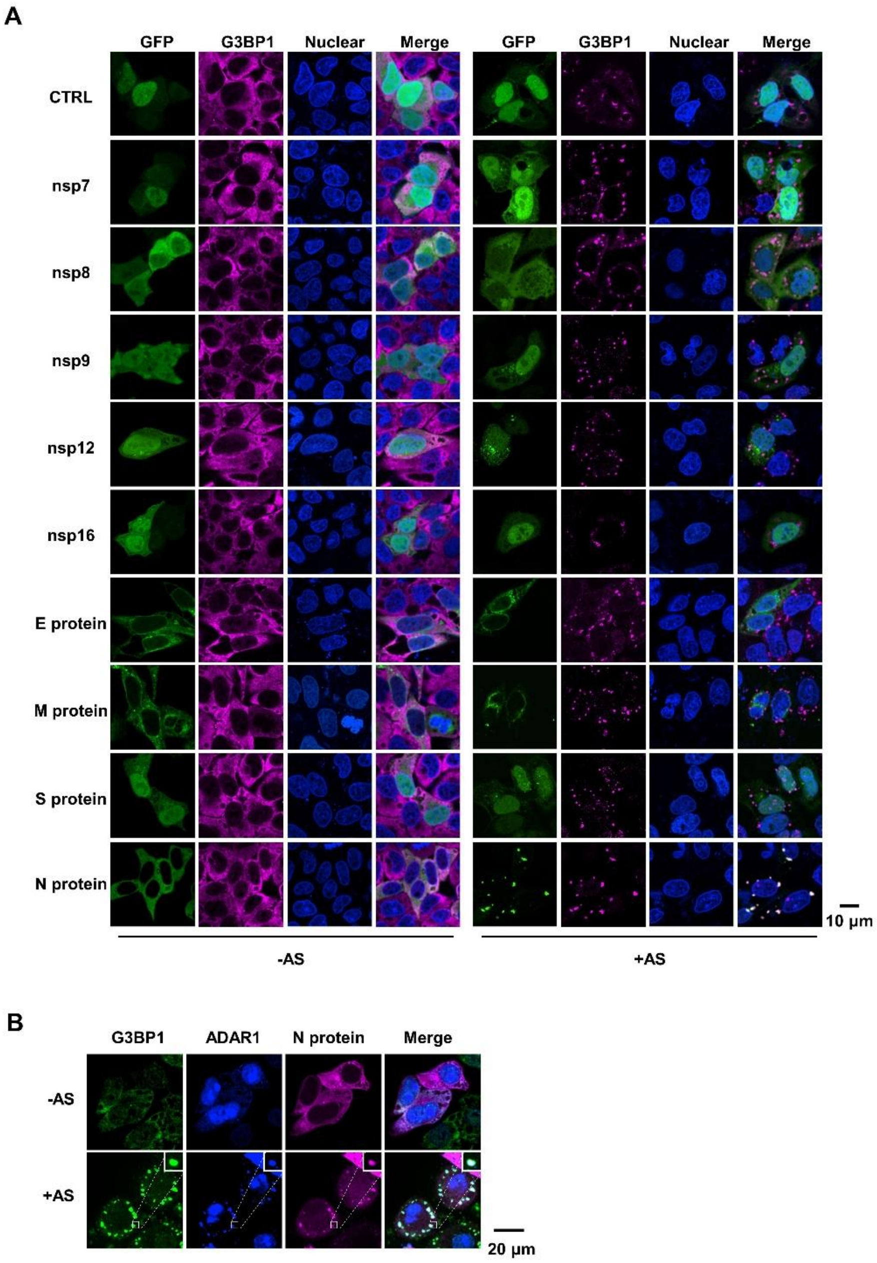

Appendix Figure S5. The SARS-CoV-2 N protein specifically co-localizes with host deaminases in SGs. (A) The SARS-CoV-2 N protein co-localizes with G3BP1 in SGs. HeLa cells were transfected with plasmids encoding GFP control or GFP-tagged SARS-CoV-2 proteins, including nsp7, nsp8, nsp9, nsp12, nsp16, S, E, M and N for 48hours, followed by treatment with AS for 45

minutes to induce SG formation. Cells were then immunostained for the endogenous G3BP1. Scale bar: 10 µm. **(B)** ADAR1 co-localizes with the N protein in SGs in HeLa cells. HeLa cells transfected with the N gene and ADAR1 were treated with AS for 45 minutes to induce SG formation, followed by immunostaining for N protein, ADAR1 and G3BP1. Scale bar: 20 µm.

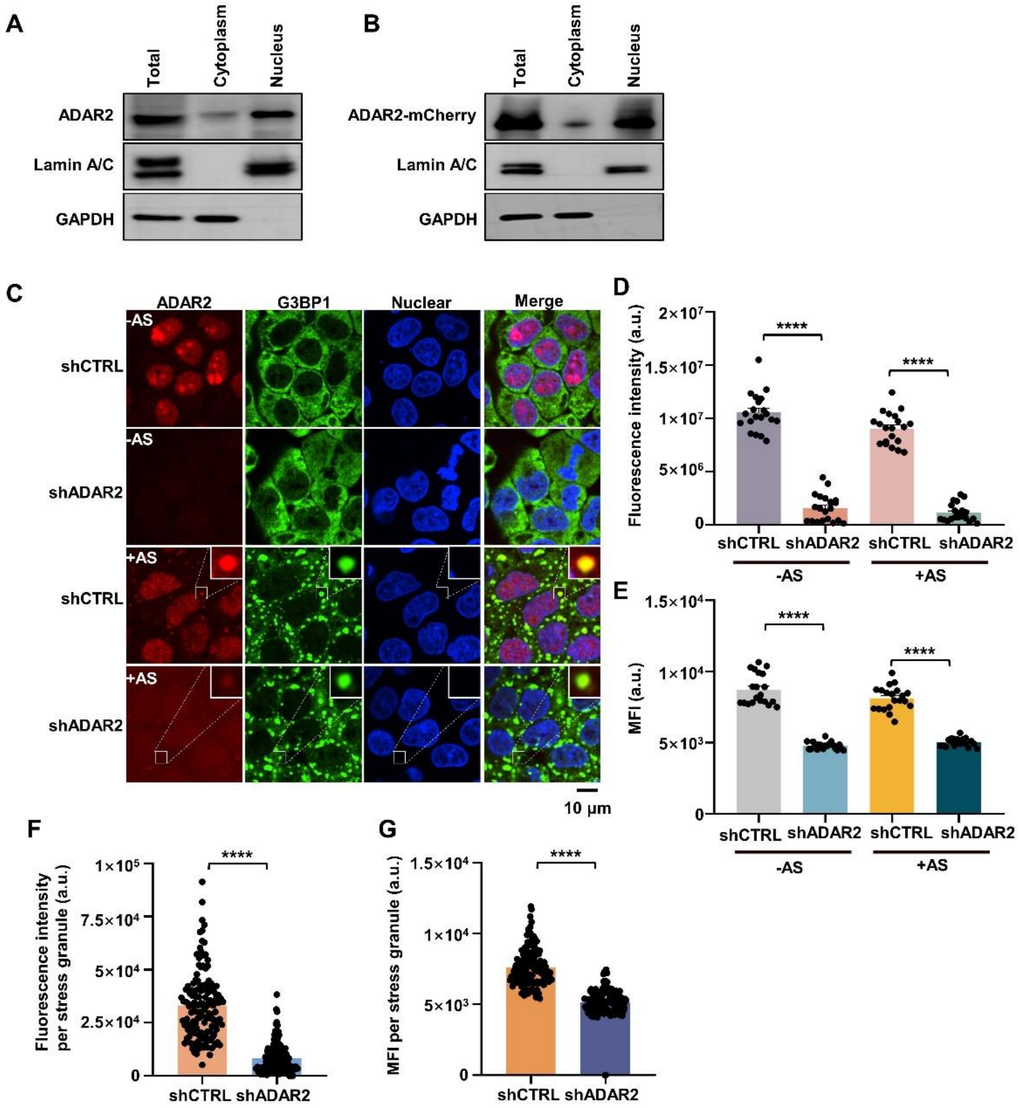

Appendix Figure S6. ADAR2 mainly localizes in both the nucleoplasm and nucleoli, and only a small fraction is present in the cytoplasm. (A) Endogenous ADAR2 distribution in the cytoplasm and nucleus. Cellular lysates were fractionated to separate the cytoplasm and the nuclei, followed by immunoprecipitation with an anti-ADAR2 antibody. GAPDH and lamin A/C proteins were used as loading controls for the cytoplasmic and nuclear fraction, respectively. **(B)** Exogenous ADAR2 distribution in the cytoplasm and nucleus. HeLa cells were transfected with plasmids encoding mCherry-tagged ADAR2. Cellular lysates were fractionated to separate the cytoplasm and the nuclei, followed by immunoprecipitation with an anti-mCherry antibody. GAPDH and lamin A/C proteins were used as loading controls in the cytoplasmic and nuclear fraction, respectively. **(C)**

ADAR2 co-localization with N protein in SGs in response to AS. HeLa cells were transfected with ADAR2-shRNA or control, and then treated with AS for 45 minutes to induce SG formation, followed by immunostaining for ADAR2 and G3BP1. Scale bar: 10 μm. **(D)-(E)** Quantitative analysis of the total fluorescence intensity (D) and mean fluorescence intensity (E) for ADAR2in relation to panel (C). Data are shown as means ± SEM (n = 20 independent images). Statistical analysis was performed with a one-way ANOVA test. ****$P < 0.0001$. **(F)-(G)** Quantitative analysis of the total fluorescence intensity (F) and mean fluorescence intensity (G) for per stress granule related to panel (C). Data are shown as means ± SEM (n = 130~140 independent stress granules). Statistical analysis was performed with a two-tailed unpaired t-test. ****$P < 0.0001$.

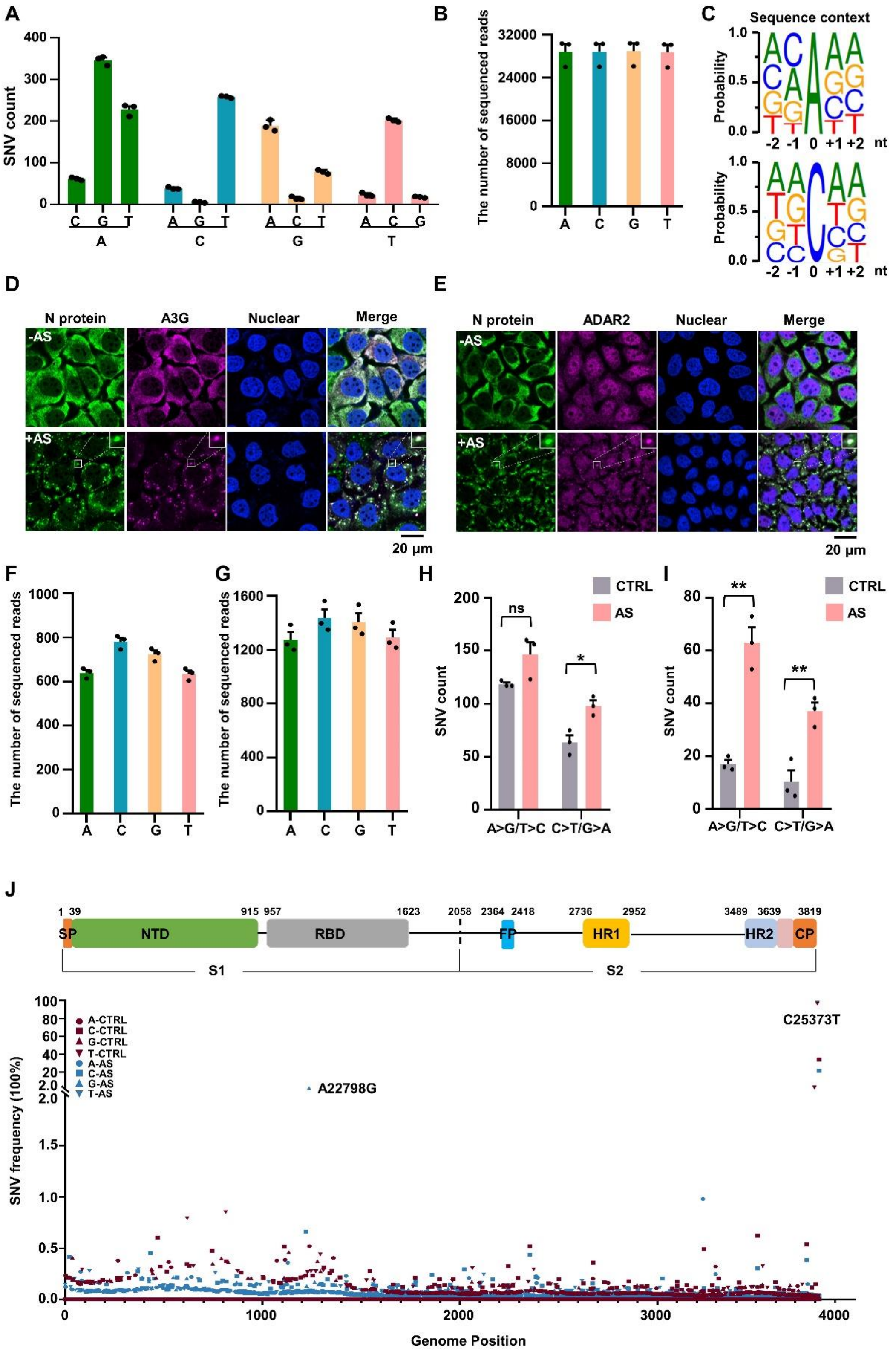

Appendix Figure S7. SARS-CoV-2 N protein co-localizes with host deaminases in SGs to facilitate viral genome mutation. (A) The number of SNVs identified in the N protein mRNA in HeLa cells with AS treatment. Values and error bars were represented as the mean ± SEM of three independent biological replicates (allelic fraction ≥0.02%). **(B)** Average sequencing read count in the N protein mRNA in HeLa cells with AS treatment. Values and error bars were represented as the

mean ± SEM of three independent biological replicates. **(C)** Sequence contexts for A>I (G) and C>U (T) edited sites in the N protein transcriptome. **(D-E)** A3G (D) or ADAR2 (E) co-localizes with the N protein in SGs in response to AS. Caco-2 cells co-transfected with the N gene and A3G or ADAR2 were treated with AS for 45 minutes to induce SG formation, followed by immunostaining for the N protein, A3G or ADAR2 and G3BP1. Scale bar: 20 µm. **(F-G)** Average sequencing read count in the SARS-CoV-2 transcriptomes in infected Caco-2 cells with (G) or without (F) AS treatment. Values and error bars were represented as the mean ± SEM of three independent biological replicates. **(H-I)** Formation of N protein-deaminase complex-involved condensates increases the number of SNVs identified in the full-length (H) or NTD and RBD (I) of spike protein in HeLa cells. Values and error bars were represented as the mean ± SEM of three independent biological replicates (allelic fraction ≥ 0.1%). Statistical analysis was performed with a two-tailed unpaired t-test. ns > 0.05, *P < 0.05, **P < 0.01. ns, no significant. **(J)** Distribution of SNVs across the full-length spike protein with or without N protein entry into deaminase-involved RNA granules. Top, schematic domain structure of spike gene. Bottom, fraction distribution of all analyzed sites across the genome of spike gene.

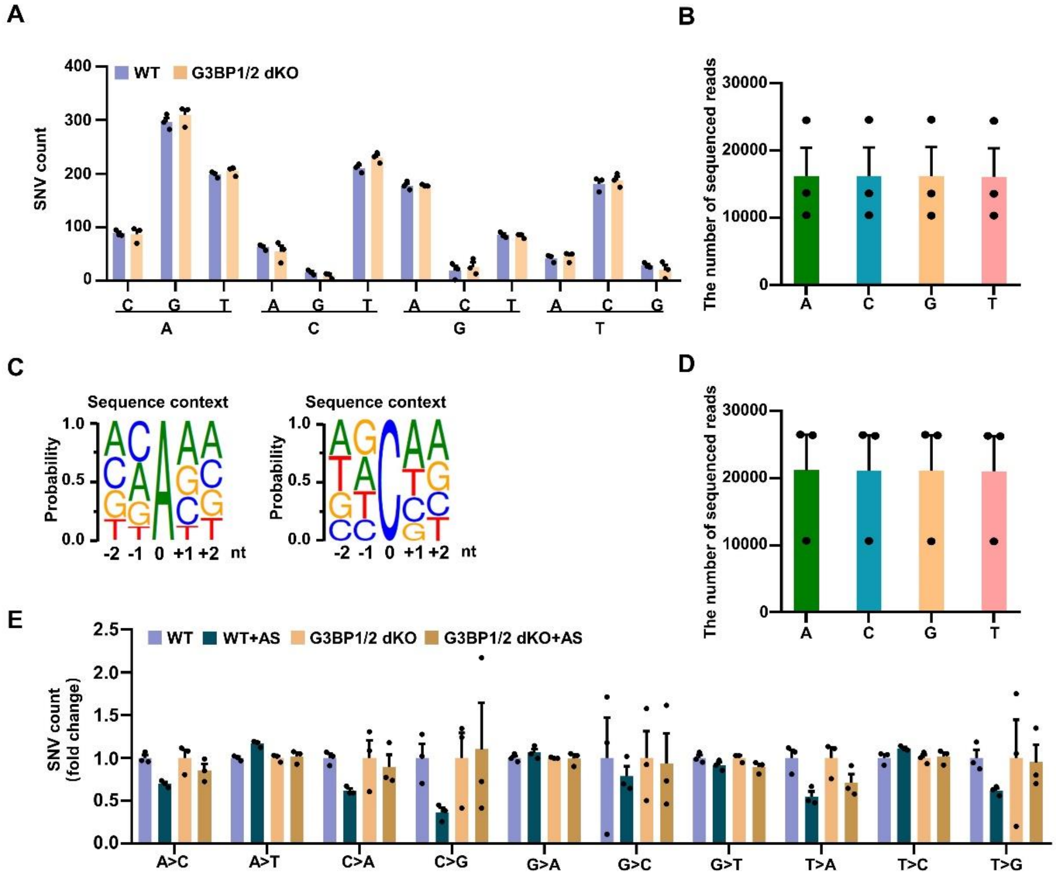

Appendix Figure S8. G3BP1/2 deletion impairs the formation of N protein-deaminase complex-involved RNA condensates and then fails to increase deaminase activity. (A) The number of SNVs identified in the N protein mRNA in HeLa cells with G3BP1/2 dKO. Data are

shown as mean ± SEM (n = 3 independent biological replicates, allelic fraction ≥0.02%). **(B)** Average sequencing read count -s in the N protein mRNA in HeLa cells with G3BP1/2 dKO. Data are shown as mean ± SEM (n = 3 independent biological replicates). **(C)** Sequence contexts for A>I (G) and C>U (T) edited sites in the N protein transcriptome. **(D)** Average sequencing read count in the N protein mRNA in G3BP1/2-null HeLa cells under AS treatment. Data are shown as mean ± SEM (n = 3 independent biological replicates). **(E)** Fold change in the number of SNVs in the N protein transcriptomes in G3BP1/2-null HeLa cells with or without AS treatment. Values and error bars were represented as the mean ± SEM of three independent biological replicates.

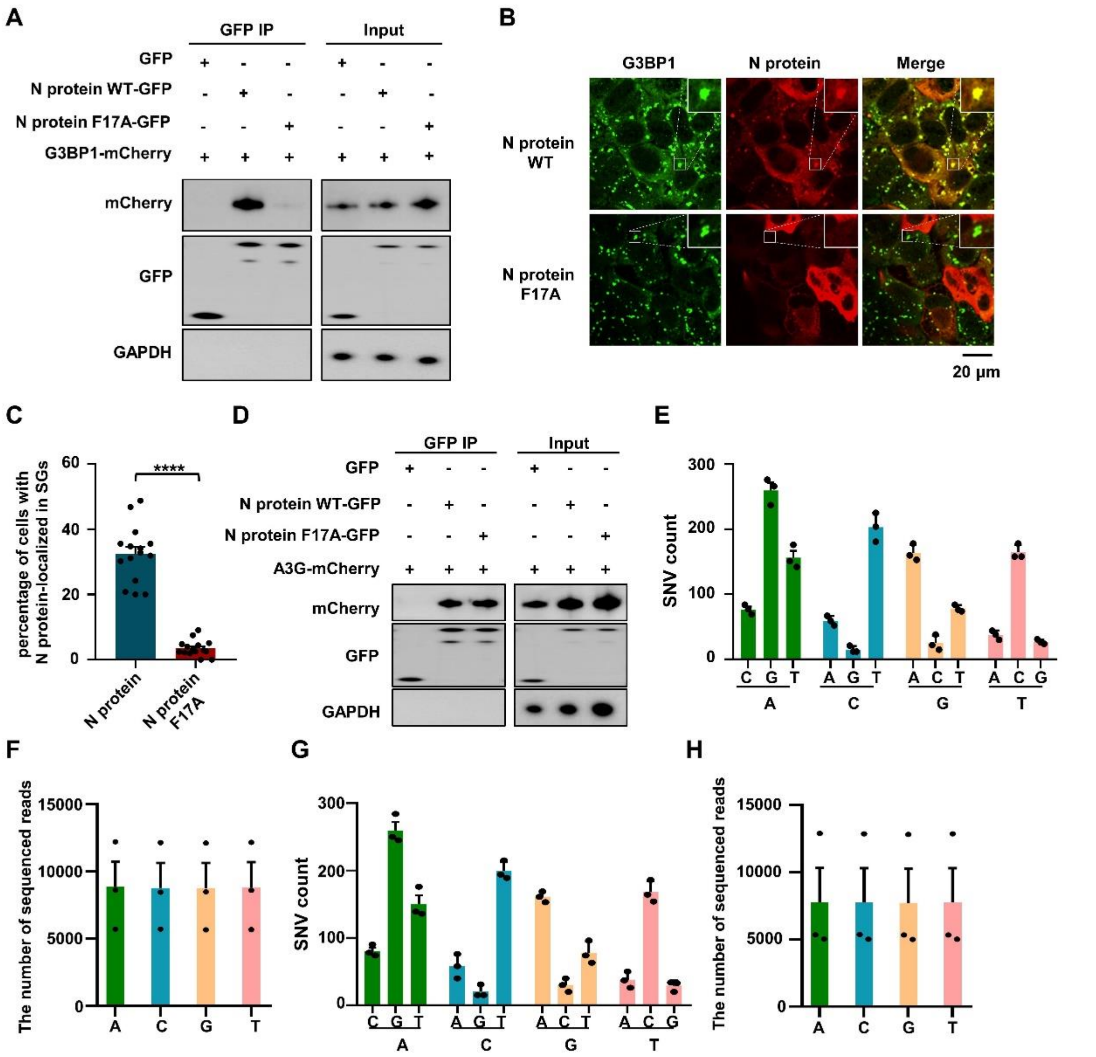

Appendix Figure S9. The N^{F17A} protein fails to interact with G3BP1 and penetrate into SGs. **(A)** The N^{F17A} protein loses the ability to interact with G3BP1. HeLa cells were co-transfected with plasmids encoding GFP-tagged N protein or N^{F17A} protein and mCherry-tagged G3BP1. Cell lysates were subjected to immunoprecipitation using an anti-GFP antibody, and the associated proteins were analyzed by western blotting. **(B-C)** The N^{F17A} protein fails to penetrate into SGs. HeLa cells co-transfected with WT N or N^{F17A} mutant were treated with AS for 45 minutes to induce SGs, followed by immunostaining for N protein (red) and the endogenous G3BP1 (green). Values and error bars

were represented as the mean ± SEM of 20 independent images. Statistics: two-tailed unpaired *t*-test. ****P < 0.0001. ns, no significant. **(D)** The N^{F17A} protein retains the ability to interact with A3G. HeLa cells were co-transfected with plasmids encoding GFP-tagged WT N or N^{F17A} mutant and mCherry-tagged A3G. Cell lysates were immunoprecipitated with an anti-GFP antibody, and the bound proteins were then analyzed by western blotting. **(E)** The number of SNVs identified in the N^{F17A} protein mRNA in HeLa cells. Data are shown as mean ± SEM (n = 3 independent biological replicates, allelic fraction ≥0.02%). **(F)** Average sequencing read count in the N^{F17A} protein mRNA in HeLa cells. **(G)** The number of SNVs identified in the $N^{\mathbf{F17A}}$ protein mRNA in HeLa cells with AS treatment. Data are shown as mean ± SEM (n = 3 independent biological replicates, allelic fraction ≥0.02%). **(H)** Average sequencing read count in the N^{F17A} protein mRNA in HeLa cells with AS treatment. Data are shown as mean ± SEM (n = 3 independent biological replicates).

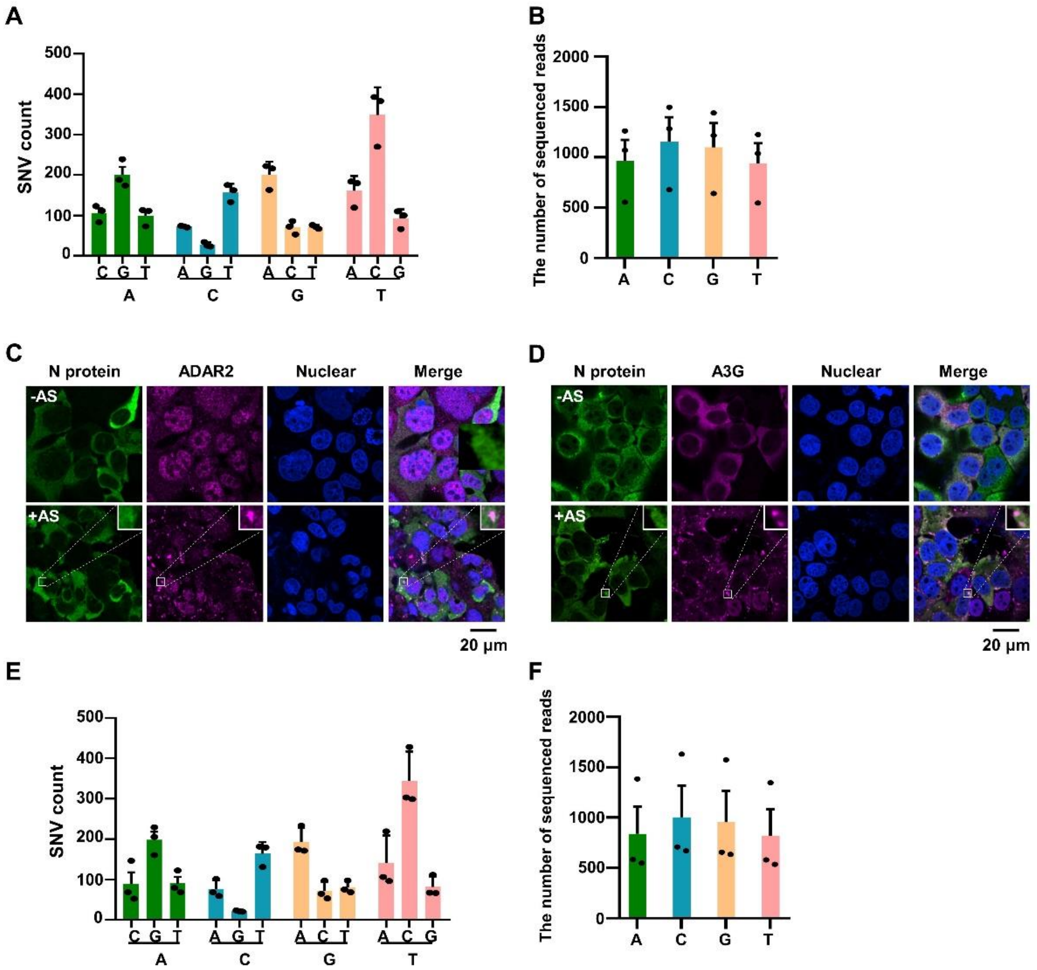

Appendix Figure S10. The N^{F17A} protein fails to enter into deaminase-localized SGs and lacks the ability to increase RNA mutation in the SARS-CoV-2 genome. **(A)** The number of SNVs identified in SARS-CoV-2 transcriptomes using a SARS-CoV-2 N^{F17A} protein-based genetic complementation system. Data are shown as mean ± SEM (n = 3 independent biological replicates, allelic fraction ≥0.1%). **(B)** Average sequencing read count in SARS-CoV-2 transcriptomes. Data

are shown as mean ± SEM (n = 3 independent biological replicates). **(C-D)** The N^{F17A} protein fails to penetrate into deaminase-enriched SGs. Caco-2 cells co-transfected with the N^{F17A} mutant were treated with AS for 45 minutes to induce SGs, followed by immunostaining for N protein and ADAR2 (C) or A3G (D). **(E)** The number of SNVs identified in SARS-CoV-2 transcriptomes using a SARS-CoV-2 N^{F17A} protein-based genetic complementation system in infected cells treated with AS. Data are shown as mean ± SEM (n = 3 independent biological replicates, allelic fraction ≥0.1%). **(F)** Average sequencing read count in SARS-CoV-2 transcriptomes. Data are shown as mean ± SEM (n = 3 independent biological replicates).

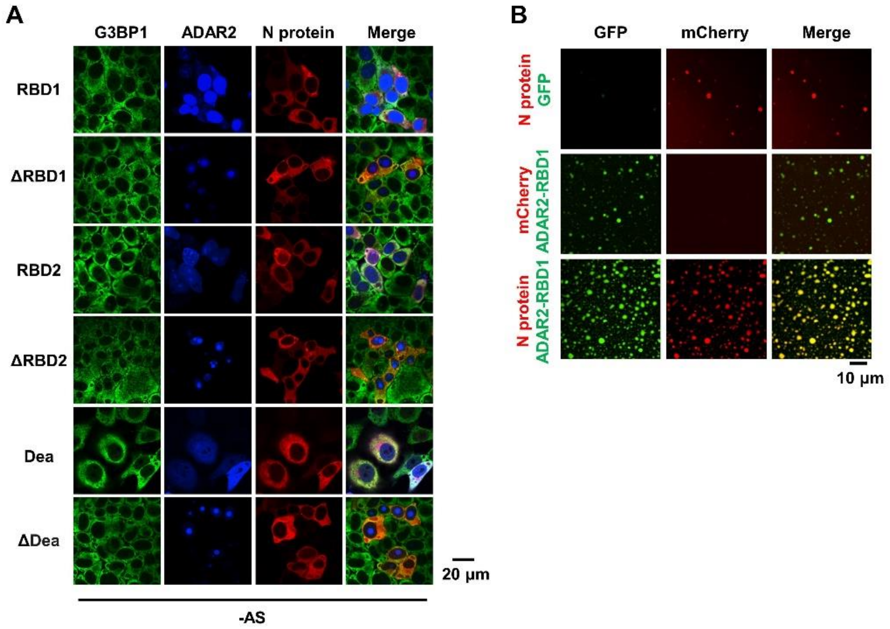

Appendix Figure S11. Analysis of ADAR2 Mutant Distribution and Phase Separation with N Protein. (A) Characterization of the subcellular distribution of ADAR2 mutants. HeLa cells were co-transfected with the N gene and BFP-tagged ADAR2 mutants, followed by immunostaining for N protein (red) and G3BP1 (green). Scale bar, 20 μm. **(B) The** N protein phase separated with ADAR2-RBD1 in an RNA-dependent manner. The concentration of each protein in reaction buffer is 2 μmol/L. Scale bar, 10 μm.

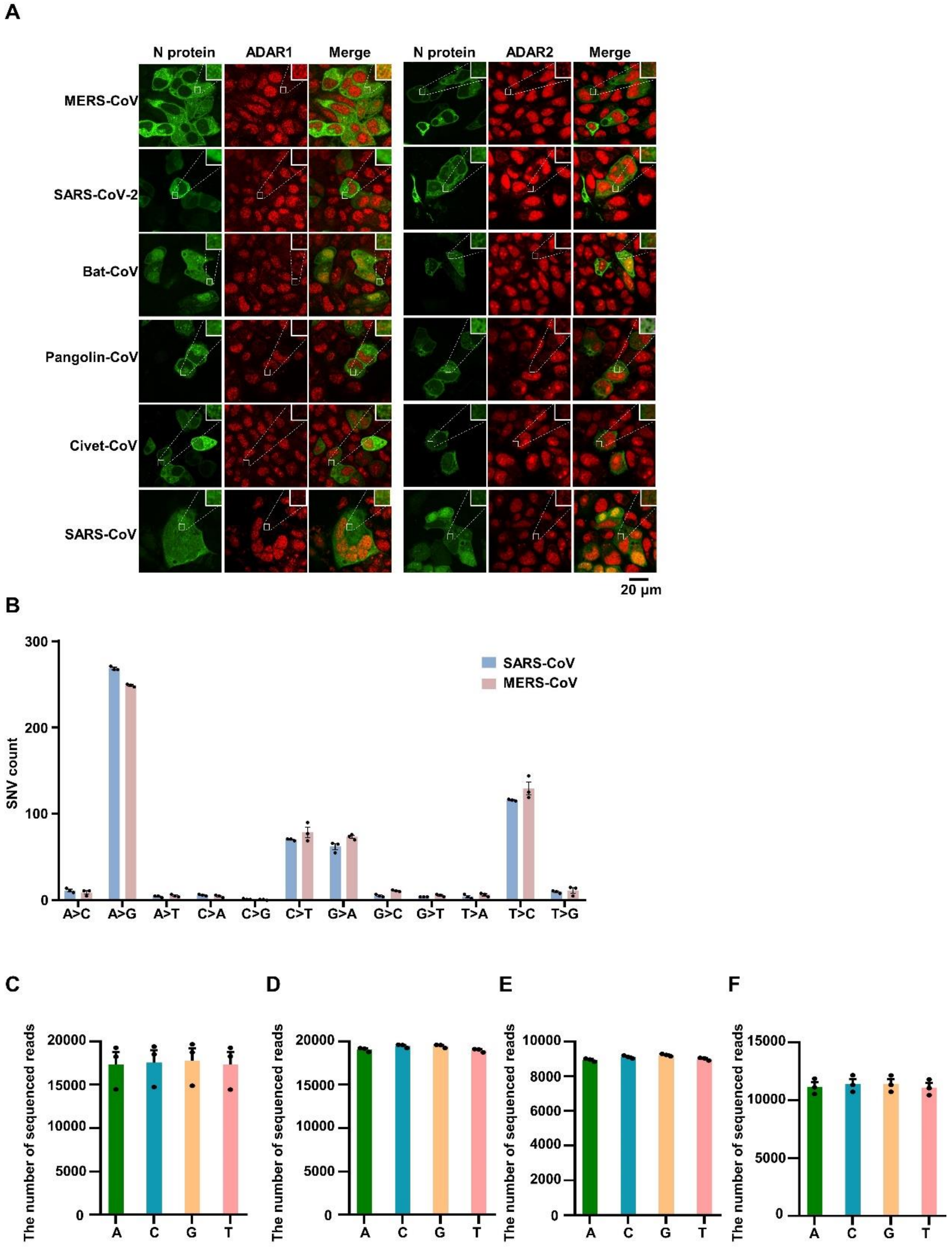

Appendix Figure S12. Functional characteristics of coronavirus N protein. (A) Depletion of G3BP1/2 abolishes the condensate formation involving ADAR1 or ADAR2-N proteins from MERS-CoV, SARS-CoV-2, bat-CoV-2, civet-CoV, pangolin-CoV and SARS-CoV. HeLa cells with G3BP1/2 dKO transfected with the various N proteins were treated with AS for 45 minutes to induce SG formation, followed by immunostaining for N protein, ADAD1 or ADAR2 and G3BP1. Scale bar: 20 μm. **(B)** Average number of SNVs in the RNA of the indicated N gene in HeLa cells. Data are shown as mean ± SEM (n = 3 independent biological replicates). **(C-D)** Average sequencing

read count in the N protein mRNA from MERS-CoV (C) and SARS-CoV (D). Data are shown as mean ± SEM (n = 3 independent biological replicates). **(E-F)** Average sequencing read count in the N protein mRNA from MERS-CoV (E) and SARS-CoV (F) in transfected cells treated with stressor. Data are shown as mean ± SEM (n = 3 independent biological replicates).
