# Supplementary figures and images for: Viral N protein hijacks deaminase-containing RNA granules to enhance SARS-CoV-2 mutagenesis
Source: EMBO J. 2024 Nov 20;43(24):6444–68. doi: 10.1038/s44318-024-00314-y (PMC11649915; doi:10.1038/s44318-024-00314-y)

Fig. 1F

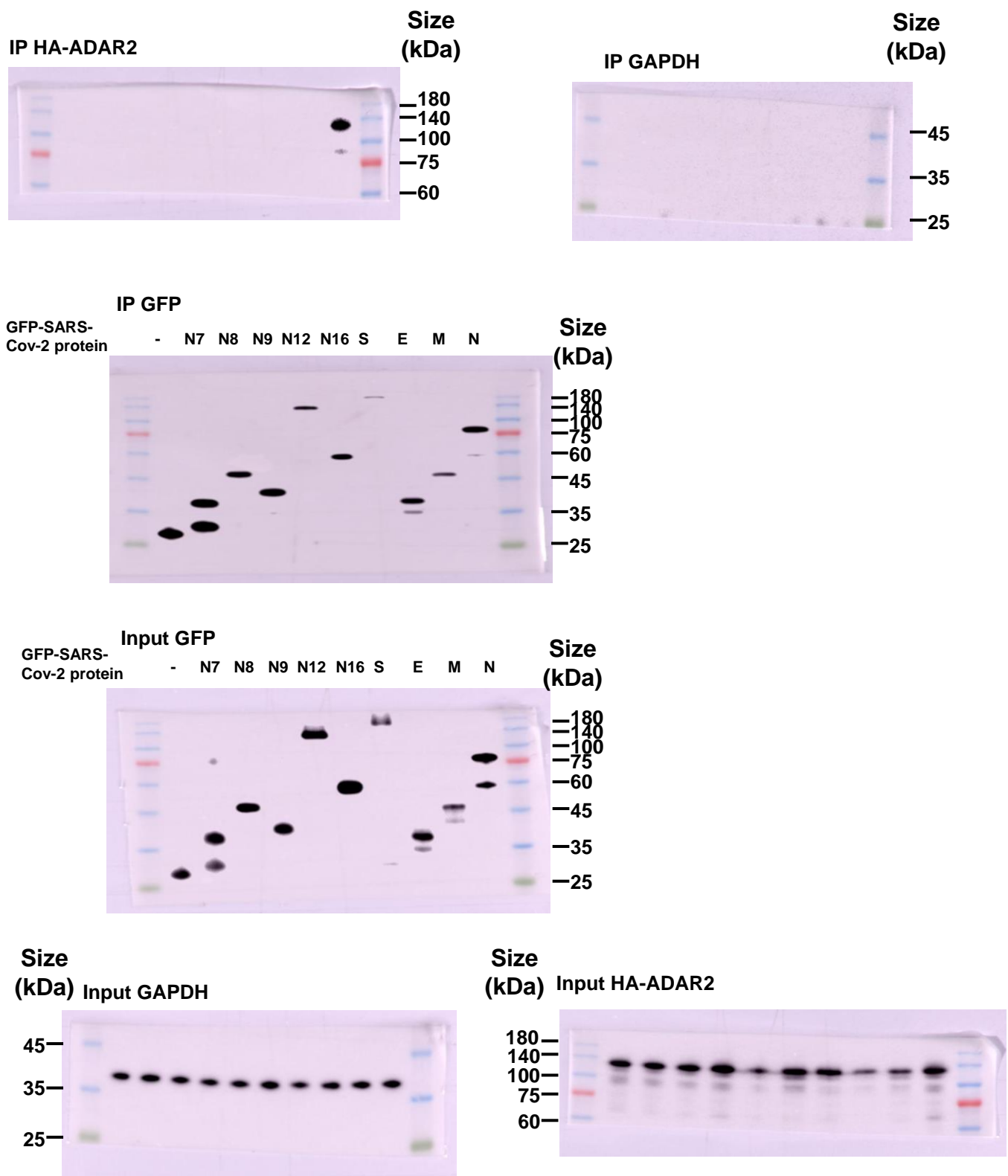

Supplement: Supplementary file 14 — Source data Fig. 1 [file 44318_2024_314_MOESM14_ESM.zip › Figure 1/1F/SourceData_Fig1F.pdf]

Fig. 1D

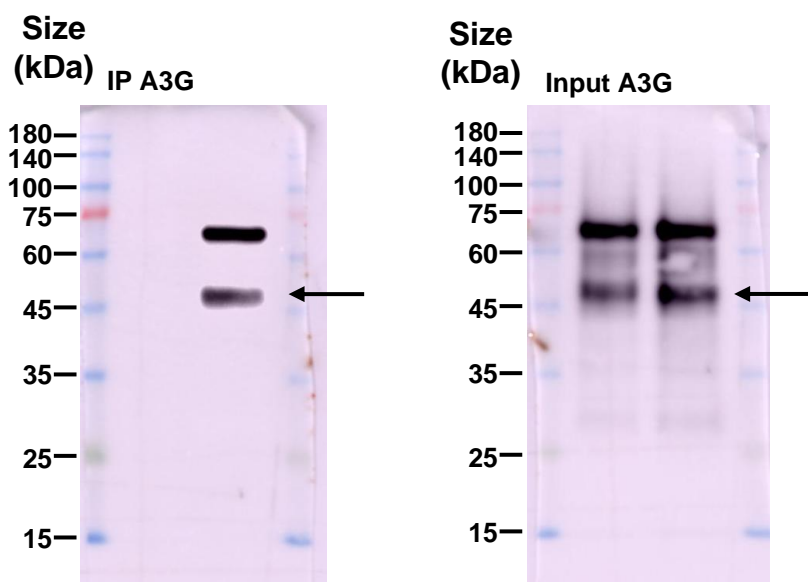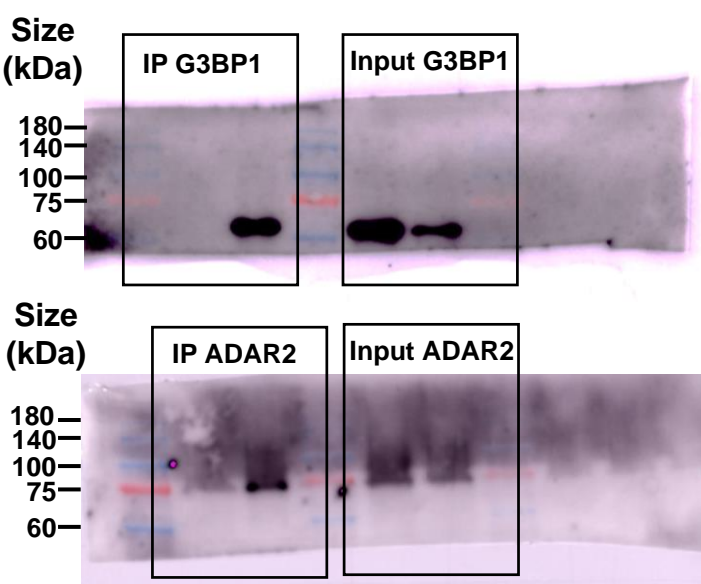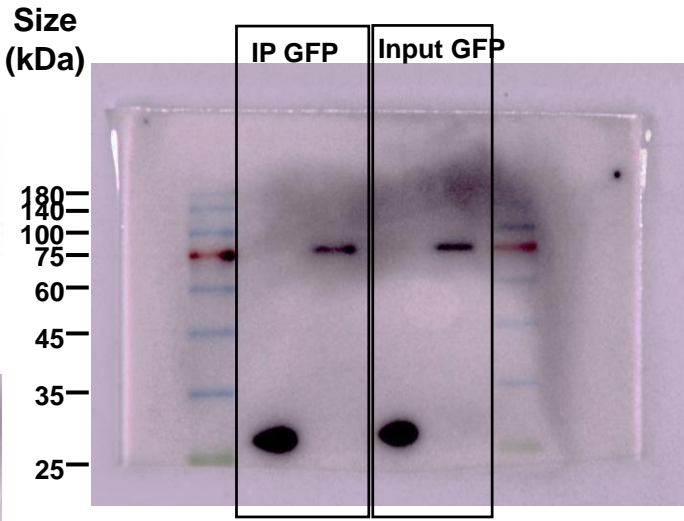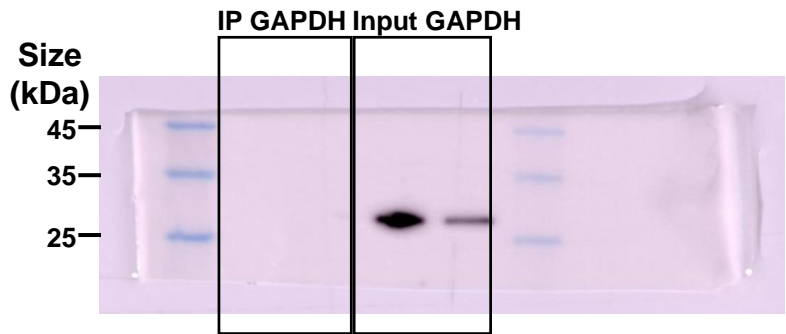

Supplement: Supplementary file 14 — Source data Fig. 1 [file 44318_2024_314_MOESM14_ESM.zip › Figure 1/1D/SourceData_Fig1D.pdf]

Fig. 2A

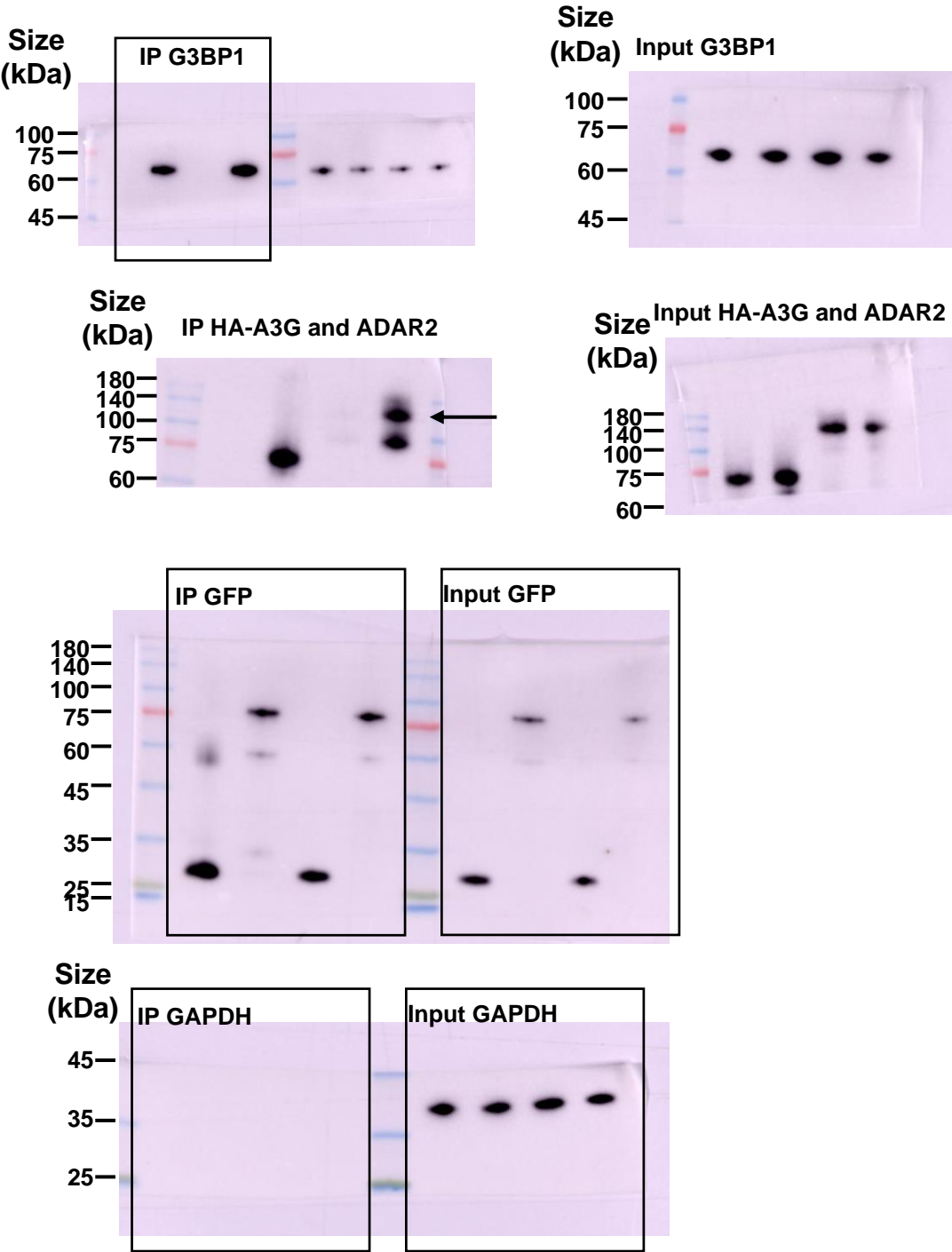

Supplement: Supplementary file 15 — Source data Fig. 2 [file 44318_2024_314_MOESM15_ESM.zip › Figure 2/2A/SourceData_Fig2A.pdf]

Fig. 2C

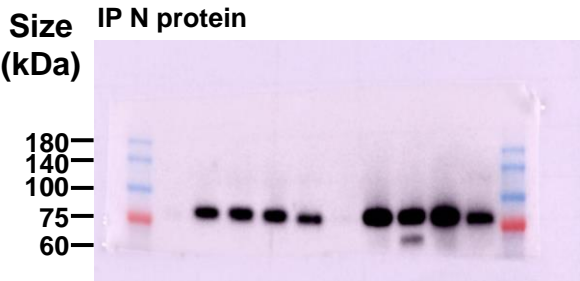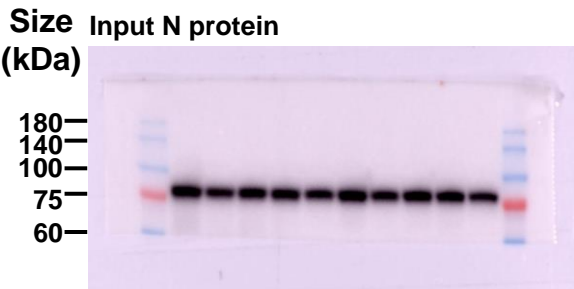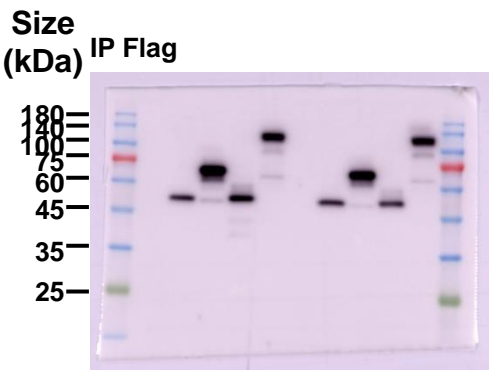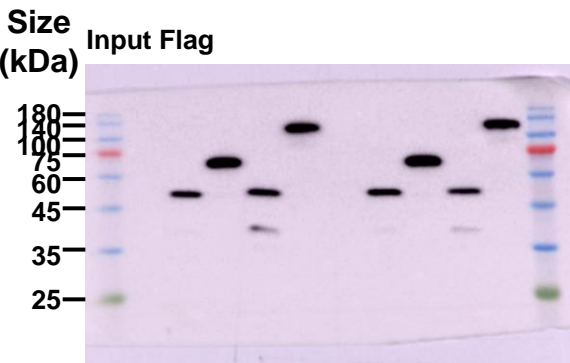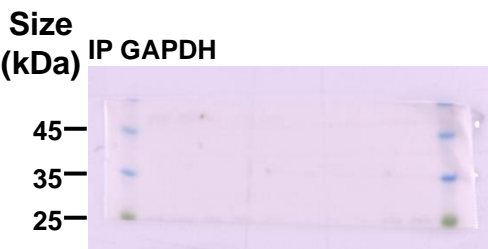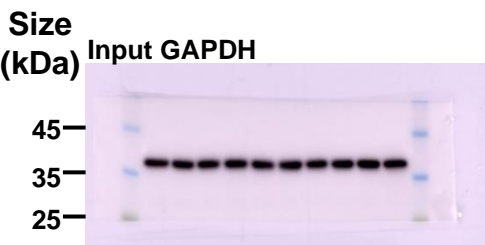

Supplement: Supplementary file 15 — Source data Fig. 2 [file 44318_2024_314_MOESM15_ESM.zip › Figure 2/2C/SourceData_Fig2C.pdf]

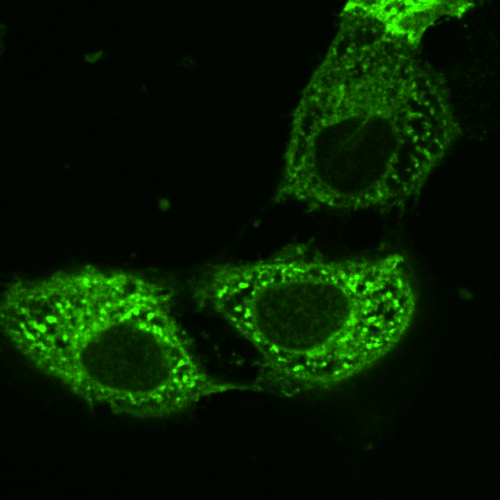

Supplement: Supplementary file 16 — Source data Fig. 3 [file 44318_2024_314_MOESM16_ESM.zip › Figure 3/3B/SourceData_Fig3B_Hela WT_sorbitol treatment_N protein.tif]

Fig 4D

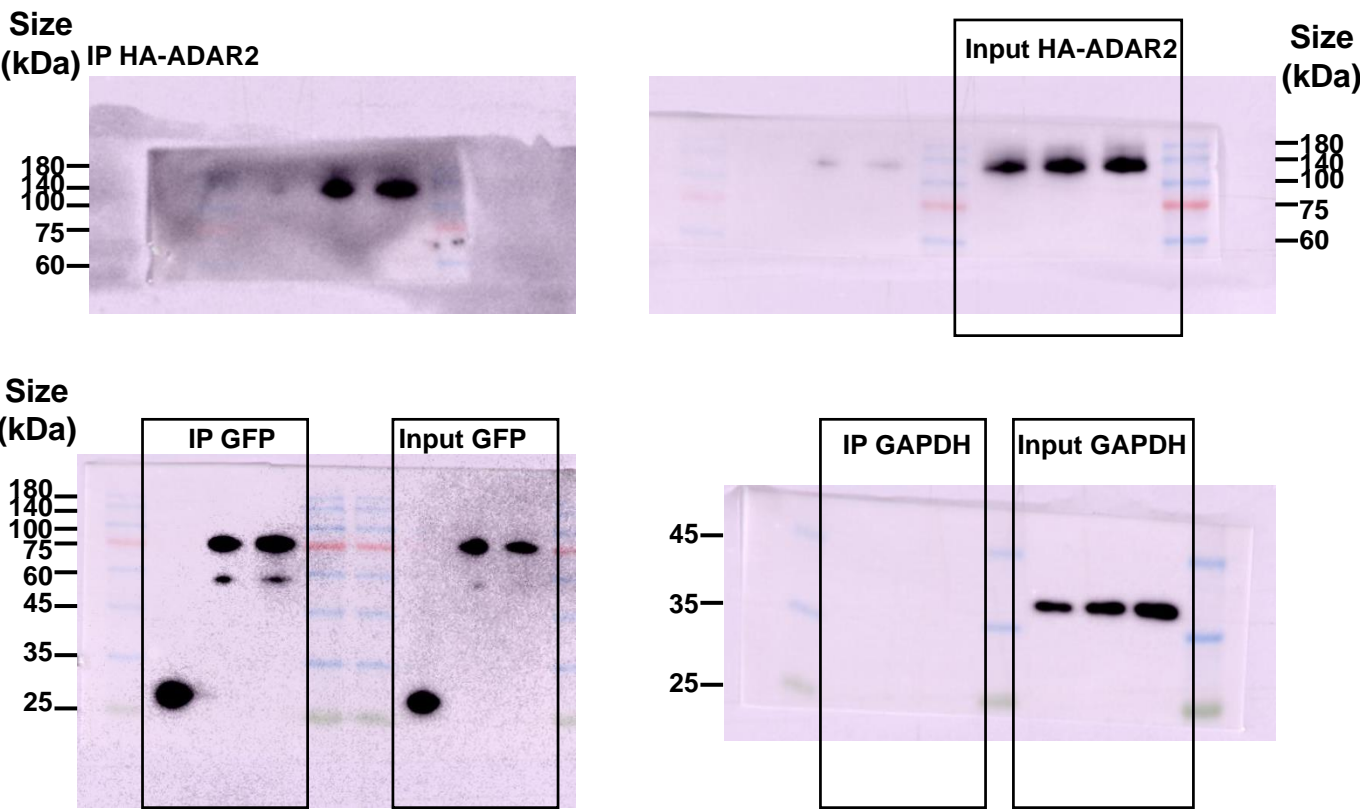

Supplement: Supplementary file 17 — Source data Fig. 4 [file 44318_2024_314_MOESM17_ESM.zip › Figure 4/4D/SourceData_Fig4D.pdf]

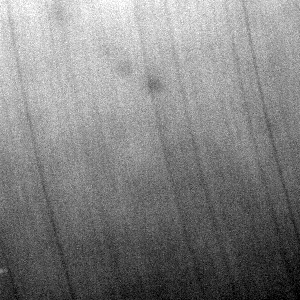

Supplement: Supplementary file 18 — Source data Fig. 5 [file 44318_2024_314_MOESM18_ESM.zip › Figure 5/5D/SourceData_Fig 5D_RNA _0s.tif]
