# Supplementary material for: Viral N protein hijacks deaminase-containing RNA granules to enhance SARS-CoV-2 mutagenesis
Source: EMBO J. 2024 Nov 20;43(24):6444–68. doi: 10.1038/s44318-024-00314-y (PMC11649915; doi:10.1038/s44318-024-00314-y)
Supplement: Supplementary file 18 — Source data Fig. 5 [file 44318_2024_314_MOESM18_ESM.zip › Figure 5/5B/SourceData_Fig5B.pdf]

Fig 5B

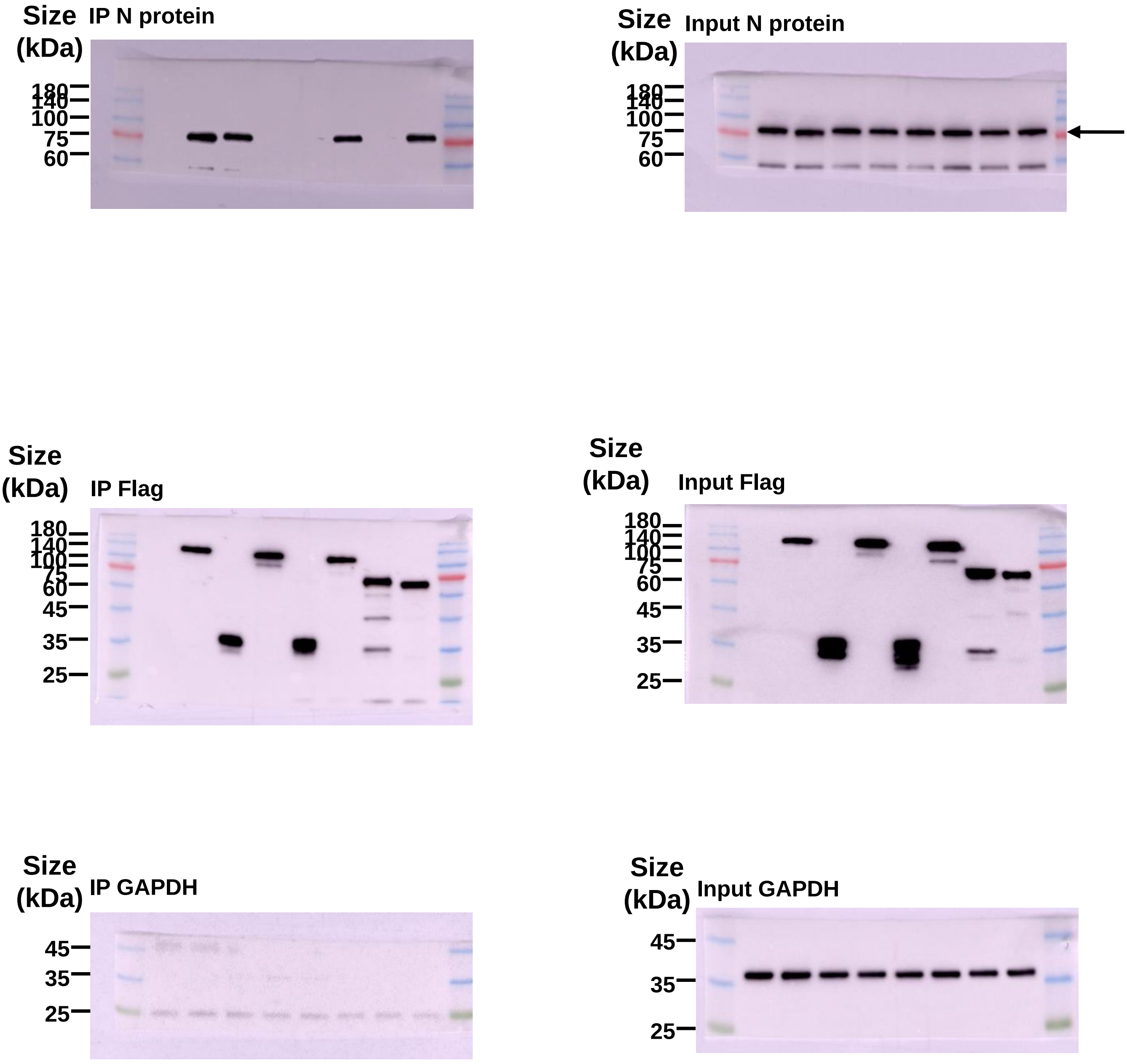
Size (kDa)
IP N protein
180
140
100
75
60
Size (kDa)
Input N protein
180
140
100
75
60
Size (kDa)
IP Flag
180
140
100
75
60
45
35
25
Size (kDa)
Input Flag
180
140
100
75
60
45
35
25
Size (kDa)
IP GAPDH
45
35
25
Size (kDa)
Input GAPDH
45
35
25
