# Supplementary material for: Viral N protein hijacks deaminase-containing RNA granules to enhance SARS-CoV-2 mutagenesis
Source: EMBO J. 2024 Nov 20;43(24):6444–68. doi: 10.1038/s44318-024-00314-y (PMC11649915; doi:10.1038/s44318-024-00314-y)
Supplement: Supplementary file 19 — Source data Fig. 6 [file 44318_2024_314_MOESM19_ESM.zip › Figure 6/6C/SourceData_Fig6C.pdf]

Fig 6C

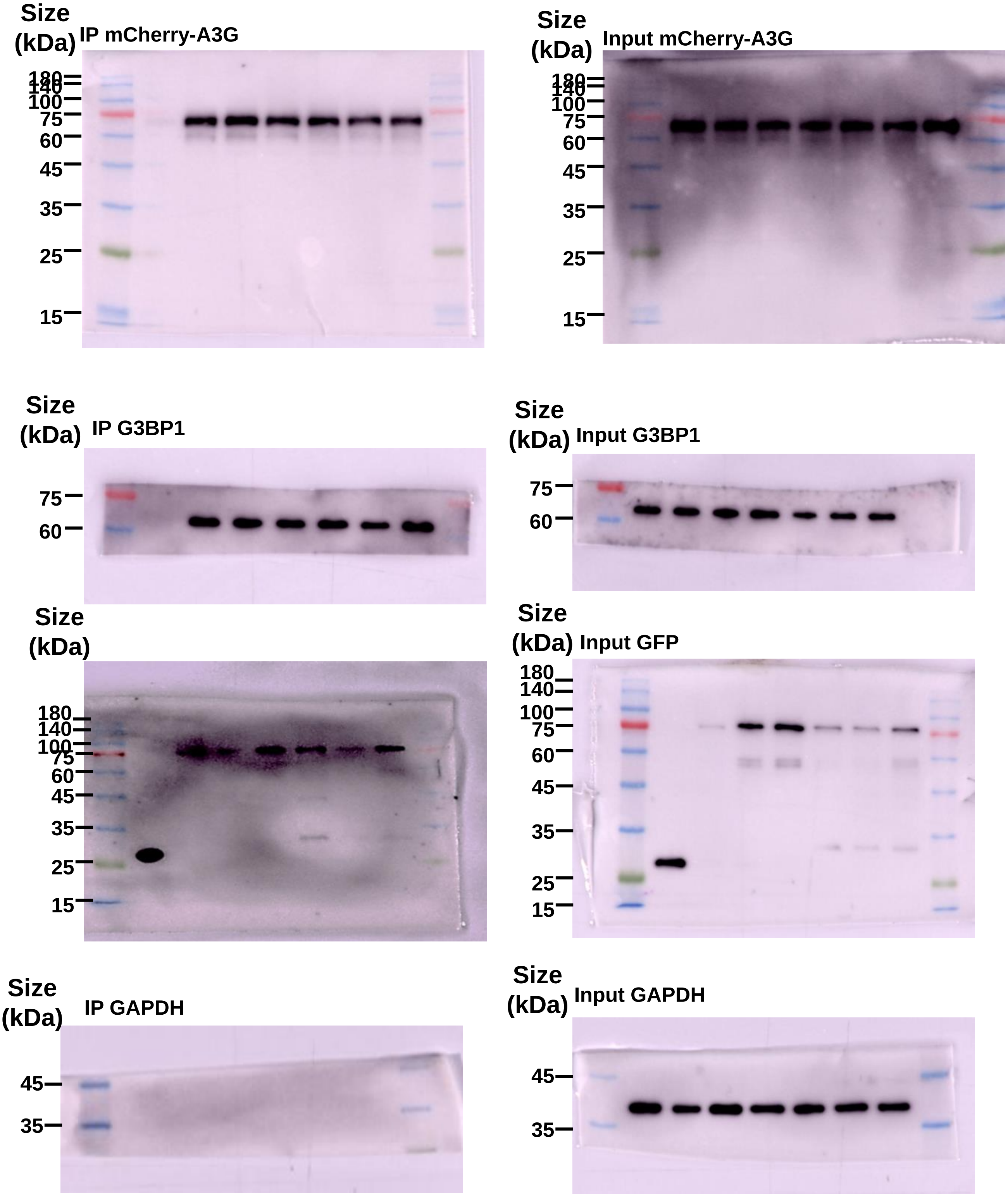

Size (kDa)
IP mCherry-A3G
180
140
100
75
60
45
35
25
15
Size (kDa)
Input mCherry-A3G
180
140
100
75
60
45
35
25
15
Size (kDa)
IP G3BP1
75
60
Size (kDa)
Input G3BP1
75
60
Size (kDa)
180
140
100
75
60
45
35
25
15
Size (kDa)
Input GFP
180
140
100
75
60
45
35
25
15
Size (kDa)
IP GAPDH
45
35
Size (kDa)
Input GAPDH
45
35
