# Supplementary material for: Viral N protein hijacks deaminase-containing RNA granules to enhance SARS-CoV-2 mutagenesis
Source: EMBO J. 2024 Nov 20;43(24):6444–68. doi: 10.1038/s44318-024-00314-y (PMC11649915; doi:10.1038/s44318-024-00314-y)
Supplement: Supplementary file 21 — Expanded View Figures [file 44318_2024_314_MOESM21_ESM.pdf]

Expanded View Figures

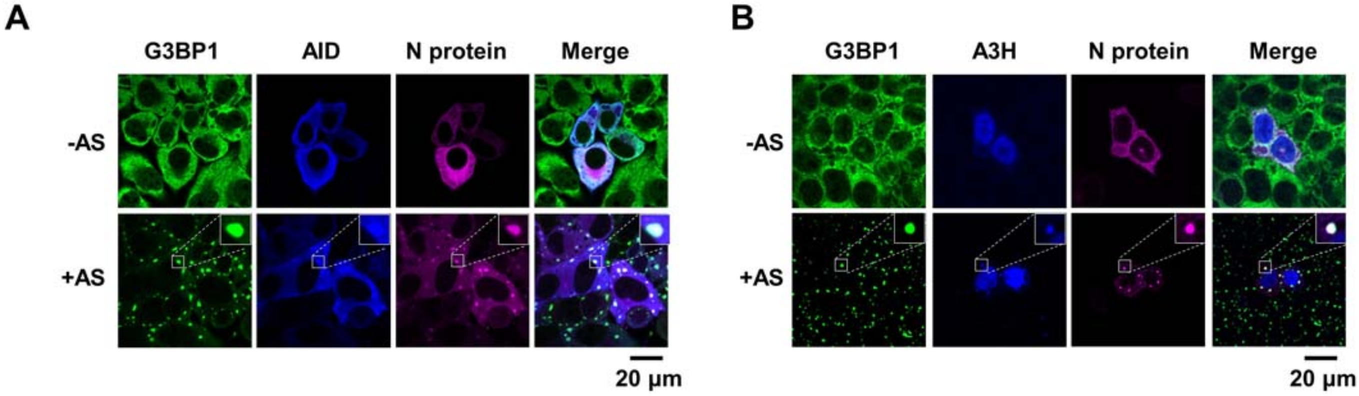

Figure EV1. Co-localization of N Protein with AID and A3H in SGs.

(**A**) AID co-localizes with N protein in SGs in HeLa cells. HeLa cells were transfected with the N gene and AID, then treated with AS for 45 min to induce SG formation, followed by immunostaining for N protein, AID and G3BP1. Scale bar: 20 μm. (**B**) A3H co-localizes with N protein in SGs in HeLa cells. HeLa cells were transfected with the N gene and A3H, then treated with AS for 45 min to induce SG formation, followed by immunostaining for N protein, A3H and G3BP1. Scale bar: 20 μm.

 © The Author(s)

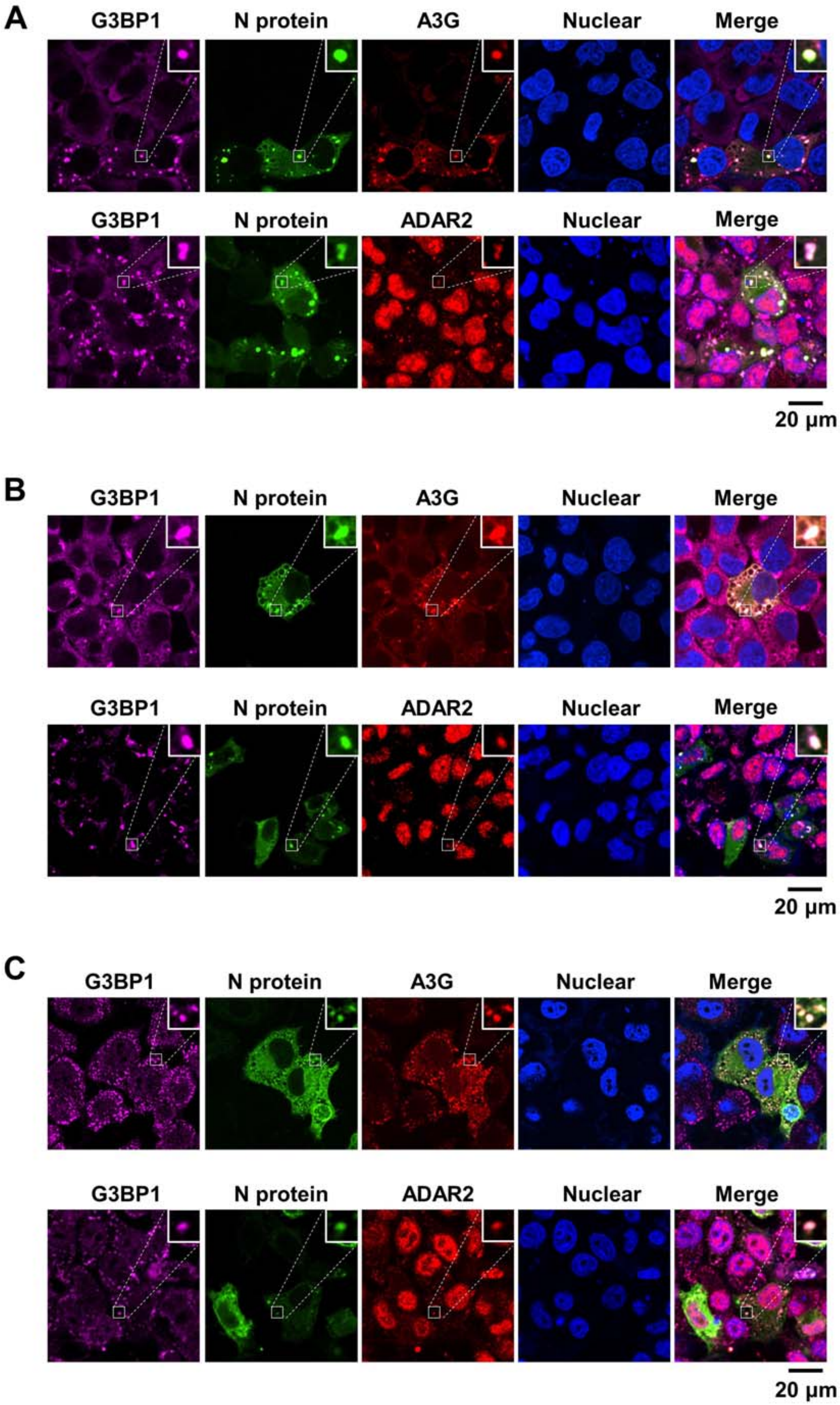
A
G3BP1
N protein
A3G
Nuclear
Merge
G3BP1
N protein
ADAR2
Nuclear
Merge
20 μm
B
G3BP1
N protein
A3G
Nuclear
Merge
G3BP1
N protein
ADAR2
Nuclear
Merge
20 μm
C
G3BP1
N protein
A3G
Nuclear
Merge
G3BP1
N protein
ADAR2
Nuclear
Merge
20 μm

Figure EV2. Specific co-localization of N protein with host deaminases under diverse stress conditions.

(**A–C**) A3G or ADAR2 co-localizes with N protein in SGs in response to various stressors. HeLa cells were transfected with the N gene and A3G or ADAR2, and treated with polyI:C for 7 h (**A**), DTT for 1 h (**B**) or sorbitol for 1 h (**C**) to induce SG formation, followed by immunostaining for N protein, A3G or ADAR2 and G3BP1. Scale bar: 20 µm.

 © The Author(s)

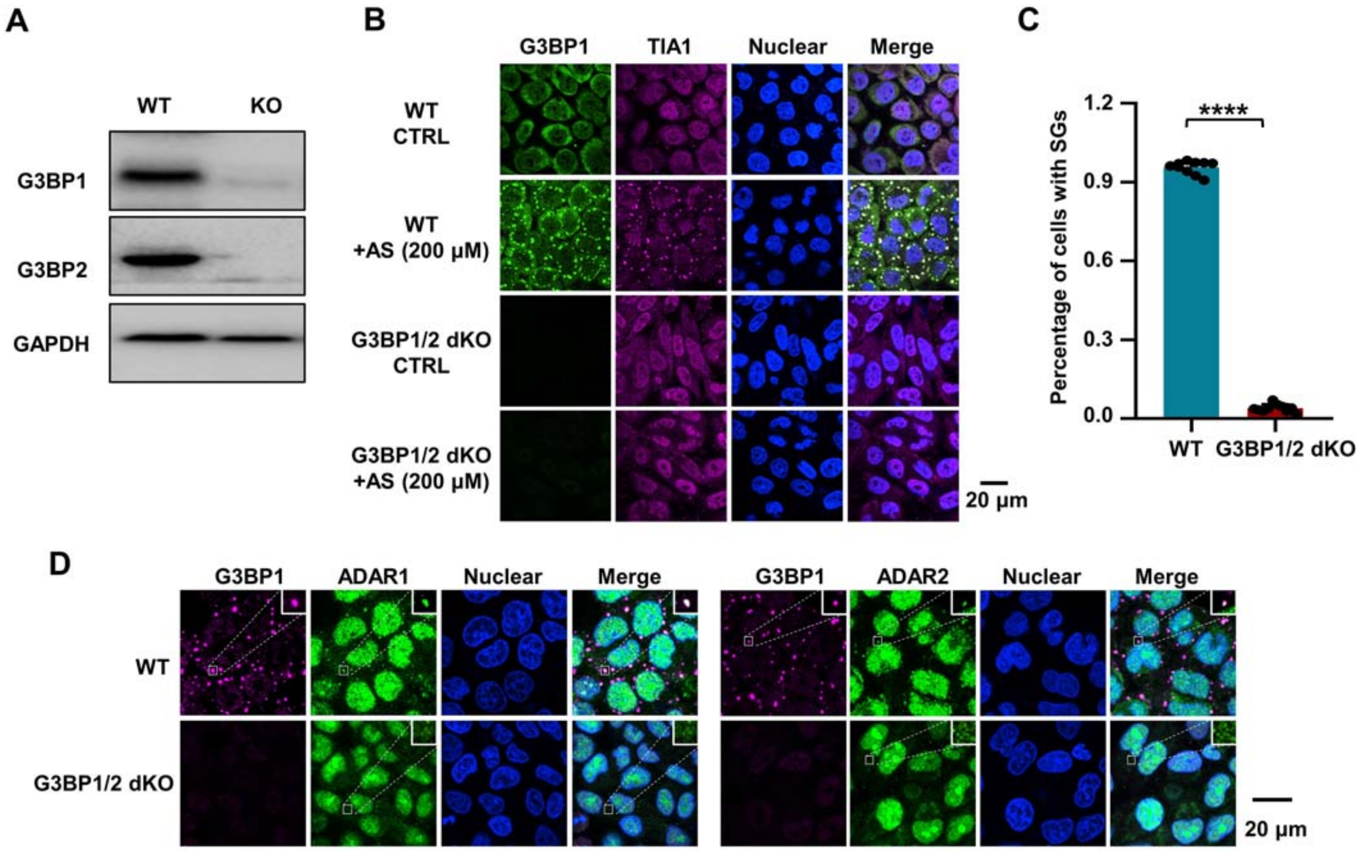

Figure EV3. The lack of G3BP1/2 disrupts the formation of N protein-deaminase complex-containing RNA condensates.

(**A**) CRISPR-Cas9-mediated G3BP1/2 knockout in HeLa cells. (**B**) G3BP1/2 dKO HeLa cells fail to form SGs under AS-induced stress. HeLa cells with or without G3BP1/2 dKO were treated with AS for 45 min to induce SG formation, followed by immunostaining for the endogenous G3BP1 (GFP) and endogenous TIA1 (red). Scale bar: 20 μm. (**C**) Quantification of SGs, expressed as the percentage of cells containing SGs in fixed HeLa cells with or without G3BP1/2 dKO. Data are shown as means ± SEM ($n = 10$ independent images). Statistical analysis was performed with a two-tailed unpaired t-test. ****$P < 0.0001$. (**D**) Depletion of G3BP1/2 abolishes the localization of ADAR1/2 in SGs under stress. HeLa cells with or without G3BP1/2 dKO were treated with AS for 45 min to induce SG formation, followed by immunostaining for endogenous G3BP1 and endogenous ADAR1 or ADAR2. Scale bar: 20 μm. Source data are available online for this figure.

© The Author(s)

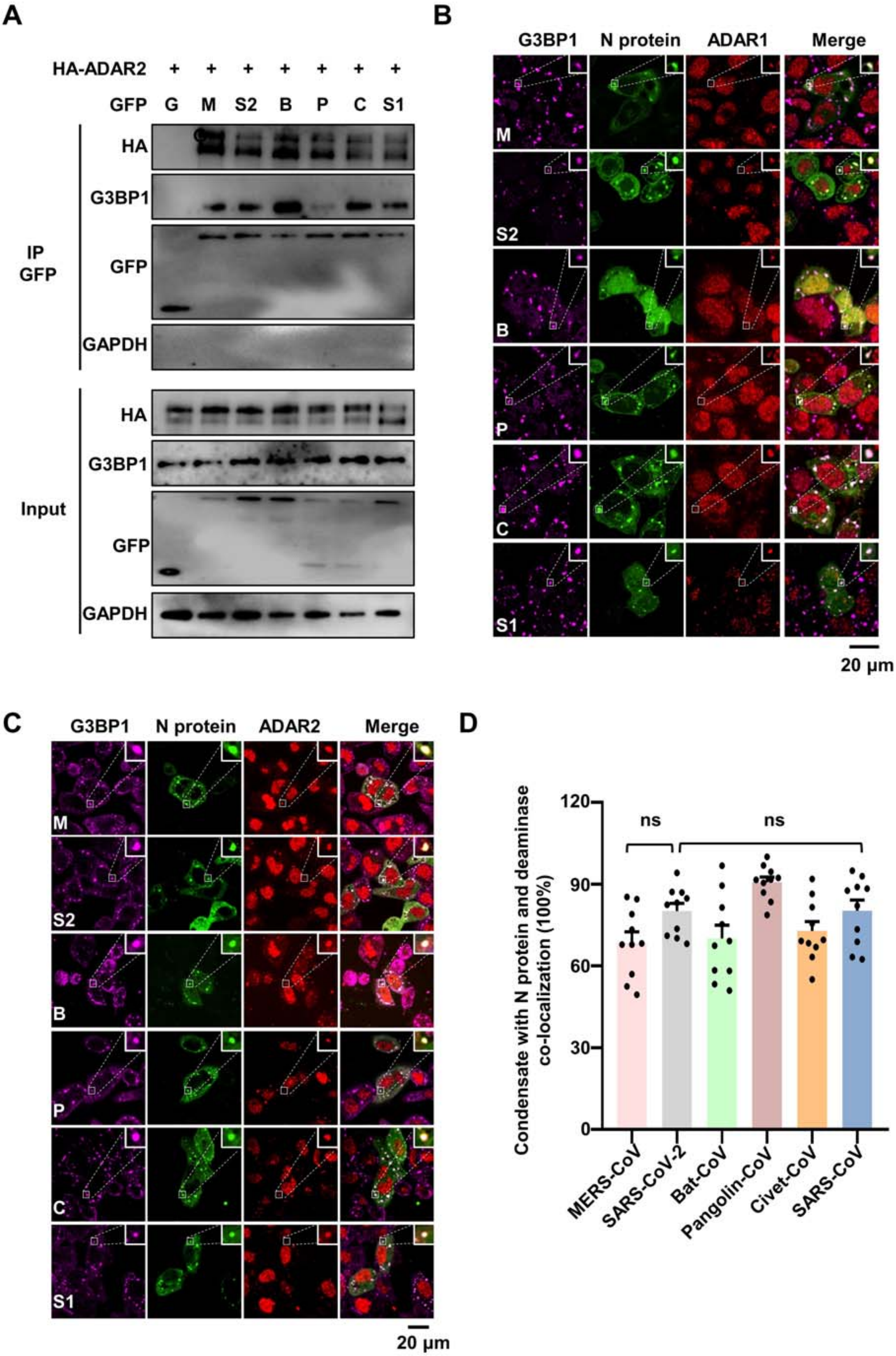
A
HA-ADAR2 + + + + + + +
GFP G M S2 B P C S1
IP GFP
HA
G3BP1
GFP
GAPDH
Input
HA
G3BP1
GFP
GAPDH
B
G3BP1 N protein ADAR1 Merge
M
S2
B
P
C
S1
20 μm
C
G3BP1 N protein ADAR2 Merge
M
S2
B
P
C
S1
20 μm
D
Condensate with N protein and deaminase co-localization (100%)
0
30
60
90
120
ns
ns
MERS-CoV
SARS-CoV-2
Bat-CoV
Pangolin-CoV
Civet-CoV
SARS-CoV

 © The Author(s)

Figure EV4. Functional characteristics of coronavirus N protein.

(**A**) Interaction assay between N protein and ADAR2. HeLa cells were co-transfected with plasmids encoding GFP-tagged N proteins of MERS-CoV, SARS-CoV-2, bat-CoV, civet-CoV, pangolin-CoV and SARS-CoV or GFP control, and HA-tagged ADAR2. Cell lysates were immunoprecipitated with an anti-GFP antibody, and the expressed proteins were analyzed by western blotting. (**B**) N proteins from MERS-CoV, SARS-CoV-2, bat-CoV, civet-CoV, pangolin-CoV and SARS-CoV exhibit the ability to co-localize with ADAR1 in SGs in HeLa cells. HeLa cells transfected with various N proteins were treated with AS for 45 min to induce SG formation, followed by immunostaining for N protein, ADAD1 and G3BP1. Scale bar: 20 µm. (**C**) N proteins from MERS-CoV, SARS-CoV-2, bat-CoV, civet-CoV, pangolin-CoV and SARS-CoV exhibit the ability to co-localize with ADAR2 in SGs in HeLa cells. HeLa cells transfected with various N proteins were treated with AS for 45 min to induce SG formation, followed by immunostaining for N protein, ADAD2 and G3BP1. Scale bar: 20 µm. (**D**) Quantification of condensates with co-localization of N protein and deaminase in fixed HeLa cells. Data are shown as mean ± SEM ($n = 10$ independent images). Statistical analysis was performed with a one-way ANOVA test. ns > 0.05, ns, not significant.
